# P023: Transfer of noroviruses between fingers and fomites

**DOI:** 10.1186/2047-2994-2-S1-P23

**Published:** 2013-06-20

**Authors:** E Duizer, E Tuladhar, W Hazeleger, M Koopmans, M Zwietering, R Beumer

**Affiliations:** 1 Department for Respiratory and Enteric Virology, Centre for Infectious Diseases Control, RIVM, Bilthoven, Netherlands; 2 Laboratory of Food Microbiology, Wageningen University, Wageningen, Netherlands; 3 Virology, Erasmus Medical Centre, Rotterdam, Netherlands

## Introduction

Human noroviruses are the major cause of acute gastroenteritis in the western world and contaminated hands are important routes for their transmission. Quantitative data on transfer during contact with surfaces and food are scarce but necessary for quantitative risk assessments. Therefore, we studied the transfer of PCR detectable units (PCRU) of human NoVs representatives of the main genogroups (GI.4 and GII.4) from fingers to fomites and vice versa. These data were compared to infectivity and PCRU data for the cultivable model murine norovirus (MNV1).

## Methods

Artificially contaminated human finger pads were pressed on stainless steel and Trespa® surfaces. In addition, clean finger pads were pressed on artificially contaminated stainless steel and Trespa® surfaces. The transfers were performed at a pressure of 0.8-1.9 kg/cm^2^ for approximately 2 s up to 7 sequential transfers either to carriers or to food products.

## Results

MNV1 infectivity transfer from finger pads to stainless steel ranged from 13± 16% on the first to 0.003± 0.009% on the fifth transfer on immediate transfer. After 10 min of drying, transfer was reduced from 0.11 ± 0.21% on the first transfer to 0.013 ± 0.023 % on the fifth transfer. MNV1 infectivity transfer from stainless steel and Trespa® to finger pads after 40 min of drying was 2.0 ± 2.0 % and 4.0 ± 5.0 % respectively.

## Conclusion

The results indicate that transfer of the virus is possible even after the virus is dried on the surface of hands or carriers. Furthermore, the role of fingers in transmission of NoVs was quantified and these data can be useful in risk assessment models and to establish target levels for efficacy of transmission intervention methods.

## Disclosure of interest

None declared

